# Inflammatory bacteriome featuring *Fusobacterium nucleatum* and *Pseudomonas aeruginosa* identified in association with oral squamous cell carcinoma

**DOI:** 10.1038/s41598-017-02079-3

**Published:** 2017-05-12

**Authors:** Nezar Noor Al-hebshi, Akram Thabet Nasher, Mohamed Yousef Maryoud, Husham E. Homeida, Tsute Chen, Ali Mohamed Idris, Newell W. Johnson

**Affiliations:** 1 Department of Maxillofacial Surgery and Diagnostic Sciences, College of Dentistry, Jazan University, Jazan, Saudi Arabia; 2 Kornberg School of Dentistry, Temple University, Philadelphia, PA USA; 3 Department of Oral and Maxillofacial Surgery, Faculty of Dentistry, Sana’a University, Yemen, Saudi Arabia; 4 Department of Microbiology, Forsyth Institute, Cambridge, MA USA; 5 Substance Abuse Research Centre (SARC), Jazan University, Jazan, Saudi Arabia; 6 Menzies Health Institute Queensland and School of Dentistry and Oral Health, Griffith University, Queensland, Australia

## Abstract

Studies on the possible association between bacteria and oral squamous cell carcinoma (OSCC) remain inconclusive, largely due to methodological variations/limitations. The objective of this study was to characterize the species composition as well as functional potential of the bacteriome associated with OSCC. DNA obtained from 20 fresh OSCC biopsies (cases) and 20 deep-epithelium swabs (matched control subjects) was sequenced for the V1-V3 region using Illumina’s 2 × 300 bp chemistry. High quality, non-chimeric merged reads were classified to species level using a prioritized BLASTN-algorithm. Downstream analyses were performed using QIIME, PICRUSt, and LEfSe. *Fusobacterium nucleatum subsp*. *polymorphum* was the most significantly overrepresented species in the tumors followed by *Pseudomonas aeruginosa* and *Campylobacter sp*. Oral taxon 44, while *Streptococcus mitis*, *Rothia mucilaginosa* and *Haemophilus parainfluenzae* were the most significantly abundant in the controls. Functional prediction showed that genes involved in bacterial mobility, flagellar assembly, bacterial chemotaxis and LPS synthesis were enriched in the tumors while those responsible for DNA repair and combination, purine metabolism, phenylalanine, tyrosine and tryptophan biosynthesis, ribosome biogenesis and glycolysis/gluconeogenesis were significantly associated with the controls. This is the first epidemiological evidence for association of *F*. *nucleatum* and *P*. *aeruginosa* with OSCC. Functionally, an “inflammatory bacteriome” is enriched in OSSC.

## Introduction

Oral cancer, predominantly squamous cell carcinoma (OSCC), is the 17^th^ most common malignant neoplasm worldwide and the 8^th^ in less developed regions^1^: it continues to have poor prognosis with 5-year survival rates less than 50% in much of the world^2, 3^. OSCC has a number of established risk factors including use of various forms of tobacco, both smoked and smokeless, drinking alcohol, human papilloma virus (HPV) infections, nutrient deficiency, solar radiation and genetic predisposition^4, 5^. However, a significant proportion of OSCC (around 15%) is not explained by these risk factors^6^, suggesting existence of other as yet unidentified risk factors worth exploring.

Recently, there has been increasing interest in the possible role of bacteria in oral carcinogenesis, inspired by the established role of some bacteria in certain types of cancer such as that of *H*. *pylori* in gastric cancer^7^. A number of studies have been carried out to this effect using various methods ranging from cultivation to 16S rRNA gene sequencing^8–20^. However, the results have been inconsistent across them. On the one hand, this is probably due to the significant methodological variations among these studies in terms of technology used for microbial analysis, type of samples obtained (biopsy, surface swab or saliva) and selection of controls (self or other subject)^7^. On the other hand, it may well be that the microbial association with OSCC is at the level of the bacterial community’s function, rather than at composition level. In other words, it may be that particular bacterial functions are associated with OSCC regardless of what species are contributing to them. In fact, a “core” functional bacteriome in the absence of a compositional one has been previously described for the gut^21^. So far, no attempt to perform functional bacteriome analysis has been made with respect to oral cancer.

The advent of next generation sequencing (NGS) has revolutionized the study of microbial communities. The 16S rRNA gene is typically targeted, enabling profiling of large number of samples at significant depth^22^ and thus detection of species which have very low abundance. Three studies have so far employed 16S rRNA-based NGS for profiling the bacteriome associated with OSCC^11, 17, 19^. However, in addition to methodological variations that hinder direct comparison of the results, these studies have used the typical bioinformatic analysis pipeline that involves *de novo* clustering of sequences into operational taxonomic units (OTUs), then using a Bayesian classifier to taxonomically assign them. This approach limits classification of the majority of sequences to the genus level^23^, rendering any associations identified of little biological significance, since specific species or even strains are usually involved in causing disease.

In a recent report, we have described a robust BLASTN-based algorithm that uses three well-curated sets of reference 16S rRNA gene sequences for classification of NGS reads to the species level, and pilot-tested it on 3 OSSC samples^10^. In the current study, we use NGS coupled with a modified version of the algorithm to profile the bacteriome within OSSC tissues in a full-scale study. In addition, we perform imputed functional analysis to predict bacterial genes and metabolic pathways associated with OSCC.

## Methods

### OSCC and control DNA samples

For cases, twenty samples were selected from an archive of anonymized, leftover DNA extracts obtained from fresh OSCC biopsies in a previous study^24^. The biopsies had been collected by Dr. Akram Nasher between June 2009 and February 2011 in 2 major hospitals in Sana’a City, Yemen as detailed in the original study, and the DNA extracts, prepared from approximately 25 mg of tissue dissected from the body of the tumors, had been stored at −80 °C since then. The selection was done so as to ensure proportional representation by gender and affected site.

Twenty healthy, gender- and aged-matched controls were recruited at the Faculty of Dentistry, Jazan University, in the South of Saudi Arabia (70 kilometers across the border with Yemen) between December 2014 and March 2015. Subjects with history of antibiotic intake in the last three months or a disease/condition known to modify oral microbial composition such as pregnancy, intake of contraceptive pills and diabetes, were excluded. Deep epithelium samples were obtained from anatomical sites matching those affected by the OSCC lesions in the cases as follows: a clean Catch-All Sample Collection swab (Epicenter, USA) was used to lightly swab the site to be sampled to remove the surface cells and adherent bacteria and then discarded. A second swab was then used to obtain deep epithelial cells by stroking with pressure 10 times in one direction, turning the swab 180° and stroking 10 times in the opposite direction. Each swab was placed in a sterile, DNAse/RNAse-free tube and stored at −20 °C.

DNA extraction from the swabs was performed using the DDK DNA isolation kit (Isohelx, UK) according to the manufacturer’s instructions. The quantity and quality of DNA, obtained from both the cases and controls, were assessed using the NanoDrop 2000 (Thermofisher Scientific, USA) and Qubit ® 2.0 Fluorimeter (Life Technologies, USA).

The study was conducted in compliance with the ethical guidelines of the Declaration of Helsinki and was approved by the biomedical research ethics committee at King Fahd University, Jazan, Saudi Arabia and an informed written consent was obtained from each of the controls. The clinical features of the cases and controls are presented in Table 1.

**Table 1:** Characteristics of the cases and control subjects included in the study (N = 40).

Variable	Cases (n = 20)	Controls(n = 20)
**Age** (mean ± SD)	53.6 ± 10.4	52.3 ± 8.9
**% males**	50	50
Site: No. (%)		
Tongue	10 (50)	9 (45)
Gum	05 (25)	5 (25)
Floor of the mouth	04 (20)	5 (25)
Buccal	01 (05)	1 (05)
**% Shammah (Arabian snuff) users**	80	15

### Amplicon library preparation and sequencing

Library preparation and sequencing were done at the Australian Centre for Ecogenomics as described previously^25^. Briefly, the degenerate primers 27FYM^26^ and 519R^27^ were used to amplify the V1-3 region of the 16S rRNA gene using standard PCR conditions. The resultant PCR amplicons (~520 bp) were then purified, indexed with unique 8-base barcodes in a 2^nd^ PCR and pooled together in equimolar concentrations. Finally, sequencing of the indexed library was performed employing the v3 2 × 300 bp chemistry on a MiSeq platform (Illumina, USA) according to the manufacturer’s protocol.

### Preprocessing of sequencing data

The raw data were submitted to Sequence Reads Archive (SRA) under project no. PRJNA352375 and preprocessed as described previously^25^. Briefly, reads with primer mismatches were removed before the primer sequences were trimmed off. The software PEAR^28^ was then employed to stitch paired sequences using the following parameters: minimum amplicon length = 432 bp; maximum amplicon lengths = 522 bp; and P-value = 0.001. Finally, the mothur software package version 1.38.1^29^ was used to process the merged reads as follows: reads with ambiguous bases, with homopolymers >8 bases long, that did not achieve a sliding 50-nucleotide Q-score average of ≥35 or that poorly aligned to SILVA reference alignment^30^ were filtered out; the remaining reads were checked for chimeras with Uchime^31^ using the self-reference approach^32^.

### Compositional data analysis

The high-quality, non-chimeric sequences were classified to the species-level employing a combination of two BLASTN-based algorithms recently described^10, 25^. Briefly, reads were individually BLASTN-searched against 4 sets of 16S rRNA reference sequences prioritized in the following order: The Human Oral Microbiome Database (HOMD) version 14.5 (available from http://homd.org/); a chimera-free version of the Human Oral Microbiome extended database (trusted-HOMDext)^10^; a modified version of the Greengene Gold set (modified-GGG)^10^; and NCBI’s Microbial 16S set (August 2016 release downloaded from ftp://ftp.ncbi.nlm.nih.gov/blast/db). Matching was done at both alignment coverage (BLASTN alignment length/read length) and percent identity (matches/alignment length) of ≥98%. Matches, if any, were first ranked by relevance (e.g. hits from HOMD 14.5 were ranked first) and then by % identity and bit score. Reads were then classified to the species level based on taxonomy of the top hit reference sequence (i.e. the sequence with the highest % identity and bit score belonging to the highest priority reference set). Reads returning top hits belonging to multiple species underwent secondary *de novo* chimera checking using USEARCH^33^ at a % identity cutoff of 98% and, if proved to be non-chimeric, were assigned multiple-species taxonomies. Reads with no matches at the specified criteria were subjected to the *de novo* chimera checking as above, and then to species-level *de novo* operational taxonomy unit (OTU) calling at 98% identity cutoff using USEARCH. Singleton OTUs were excluded and a representative sequence for each of the remaining OTUs was BLASTN-searched against the 4 reference sets again to determine the closest species for taxonomy assignment.

The QIIME (Quantitative Insights Into Microbial Ecology) software package version 1.9.1^34^ was used to perform downstream analysis including subsampling to obtain an equal number of reads across the samples, generation of taxonomy plots/tables and rarefaction curves and calculation of species richness, coverage and a range of alpha and beta diversity indices. Comparison between samples in bacterial community membership and structure was performed with Principal Component Analysis (PCoA) based on binary and weighted Jaccard distance matrices. Detection of differentially abundant taxa between the cases and controls was done using Linear discriminant analysis Effect Size (LEfSe)^35^ and G-test.

### Functional prediction analysis

The reads were reclassified with mothur using Wang’s method and Greengenes 97% OTUs (version 13.5) as reference. The reads were then assigned to OTUs based on their taxonomy (phylotype command) and the generated file was converted into a BIOM (Biological Observation Matrix) table. The latter was then used as an input to PICRUSt (phylogenetic investigation of communities by reconstruction of unobserved states)^36^, a bioinformatics resource for prediction of functional content of microbial communities by matching OTUs in the samples to reference OTUs with known/imputated gene content, normalizing for gene-copy number variations. The analysis was performed based on KEGG orthologs (KO) and pathways. Differences in genes and pathways between the cases and controls were explored using LEfSe.

## Results

### Sequencing and data processing statistics

The sequencing run generated 5,037,910 raw paired reads. Around 20% of these were identified with primer mismatches and removed. The majority of the remaining reads (89.9%) could be successfully stitched with PEAR. However, only 21.8% of the merged reads withstood the stringent quality-filtration strategy, which we previously found to reduce sequencing error rates by 10 fold^25^. An additional 180,109 reads were filtered out in subsequent alignment and chimera checks, leaving a final of 611, 225 high-quality, non-chimeric merged reads with an average length of 477 bp. Around 94% of these reads were successfully classified to the species level; 172 reads were identified de novo as additional chimeras; 184 did not return BLASTN matches and 36,573 formed singleton OTUs and were thus excluded. The number of classified reads per sample ranged from 7791 to 28152 reads (14357 ± 4499 reads per subject). The results described below were based on a minimum read count per species (MC) of one. Results for higher MC cutoffs (10 and 100) can be found at ftp://www.homd.org/publication_data/20170317/qiime_results/index.html.

### Bacteriome profile

**Figure 1:**
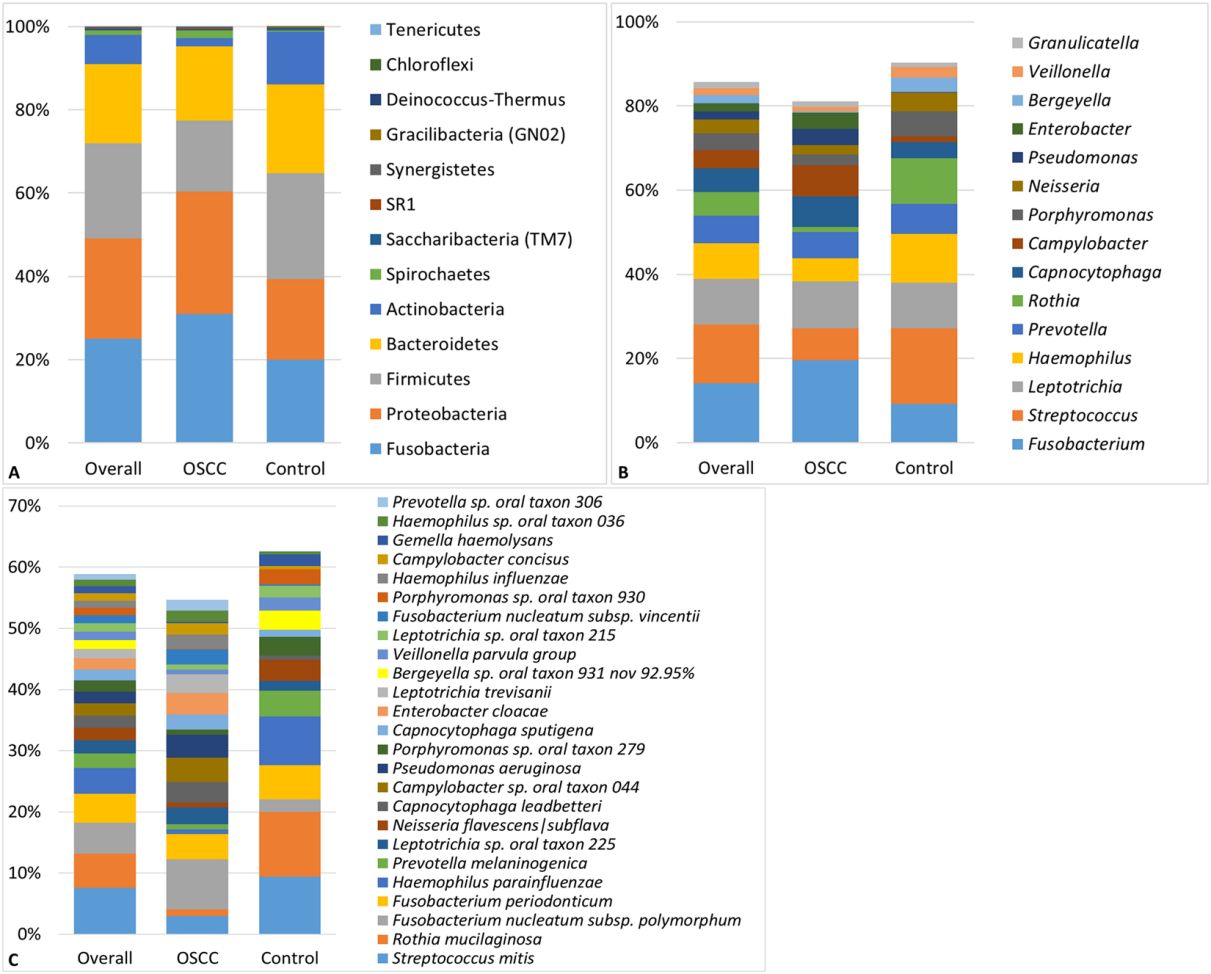
Bacteriome profile. Stacked bars showing the distribution of phyla, top 15 genera and top 25 species detected in the study population and groups.

A total of 1,118 species-level taxa, including 416 potentially novel species, belonging to 259 genera and 13 phyla were identified in the samples. The abundances and detection frequencies of these in each of the samples and across the study groups are presented in Supplementary Datasets 1–3. The number of species detected in the cases and controls was 795 and 746, respectively, with 423 species in common. Per sample, the number of species ranged from 53 to 254 and 79 to 245 for the cases and controls, respectively (average of 140 and 144 species per sample, respectively). Figure 1 displays the distribution of the phyla, top 15 genera and top 25 species detected. Overall, phyla Fusobacteria, Proteobacteria, Firmicutes, Bacteroidetes, Actinobacteria and genera *Fusobacterium*, *Streptococcus*, *Leptotrichia*, *Haemophilus*, *Prevotella*, *Rothia*, *Capnocytophaga*, *Campylobacter*, *Porphyromonas* and *Neisseria* accounted for the bulk of the bacteriome. At the species level, *Streptococcus mitis*, *Rothia mucilaginosa*, *Fusobacterium nucleatum subsp*. *polymorphum*, *Fusobacterium periodonticum*, *Haemophilus parainfluenzae*, *Prevotella melaninogenica*, *Leptotrichia sp*. *oral taxon 225*, *Neisseria flavescens|subflava* were overall the most predominant species.

**Figure 2:**
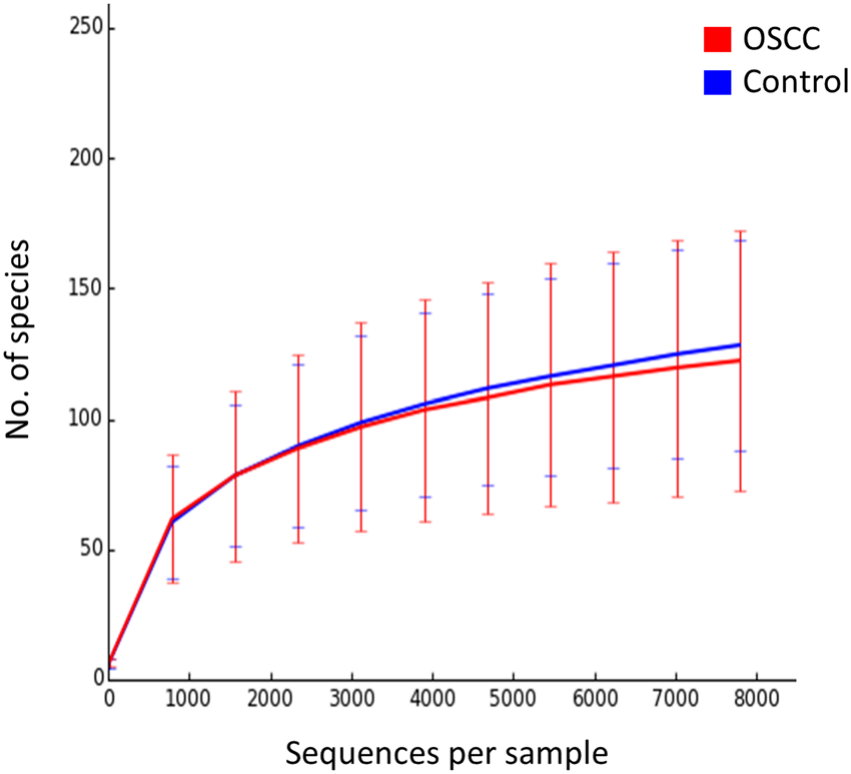
Rarefaction curves showing the number of observed species as a function of sequencing depth.

**Figure 3:**
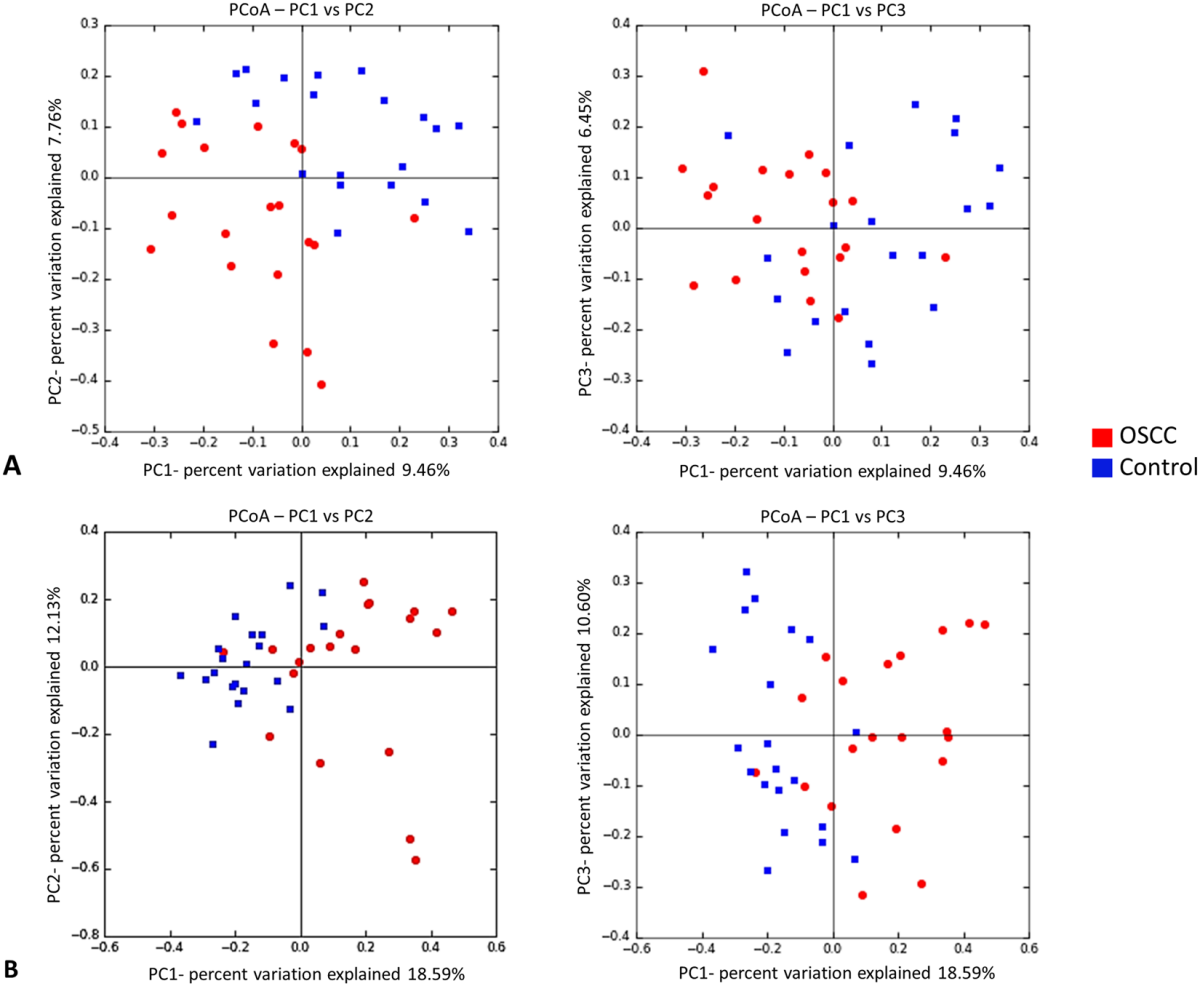
Principal Component Analysis. Clustering of the samples based on (**A**) binary Jaccard index (community membership) and (**B**) abundance weighted Jaccard index (community structure).

The two study groups were comparable in terms of species richness, α-diversity and coverage (Table 2). Rarefaction indicated sufficient sequencing depth (Fig. 2). Figure 3 shows the separation between OSCC and control samples by PCoA based on community membership (mere presence/absence of species) and structure (taking into account their relative abundances), using binary and abundance weighted Jaccard indices, respectively. More variation could be explained based on community structure.

**Table 2:** Species richness, α-diversity and coverage (mean ± SE) calculated from the rarefied biom.

Product type	Observed richness	Chao1	Shannon index	Good’s coverage
OSCC	122.2 ± 49.9	145.7 ± 59.1	4.033 ± 0.939	0.997 ± 0.002
Control	128.2 ± 40.5	161.9 ± 47.6	3.876 ± 0.997	0.996 ± 0.001

### Differentially abundant taxa

**Figure 4:**
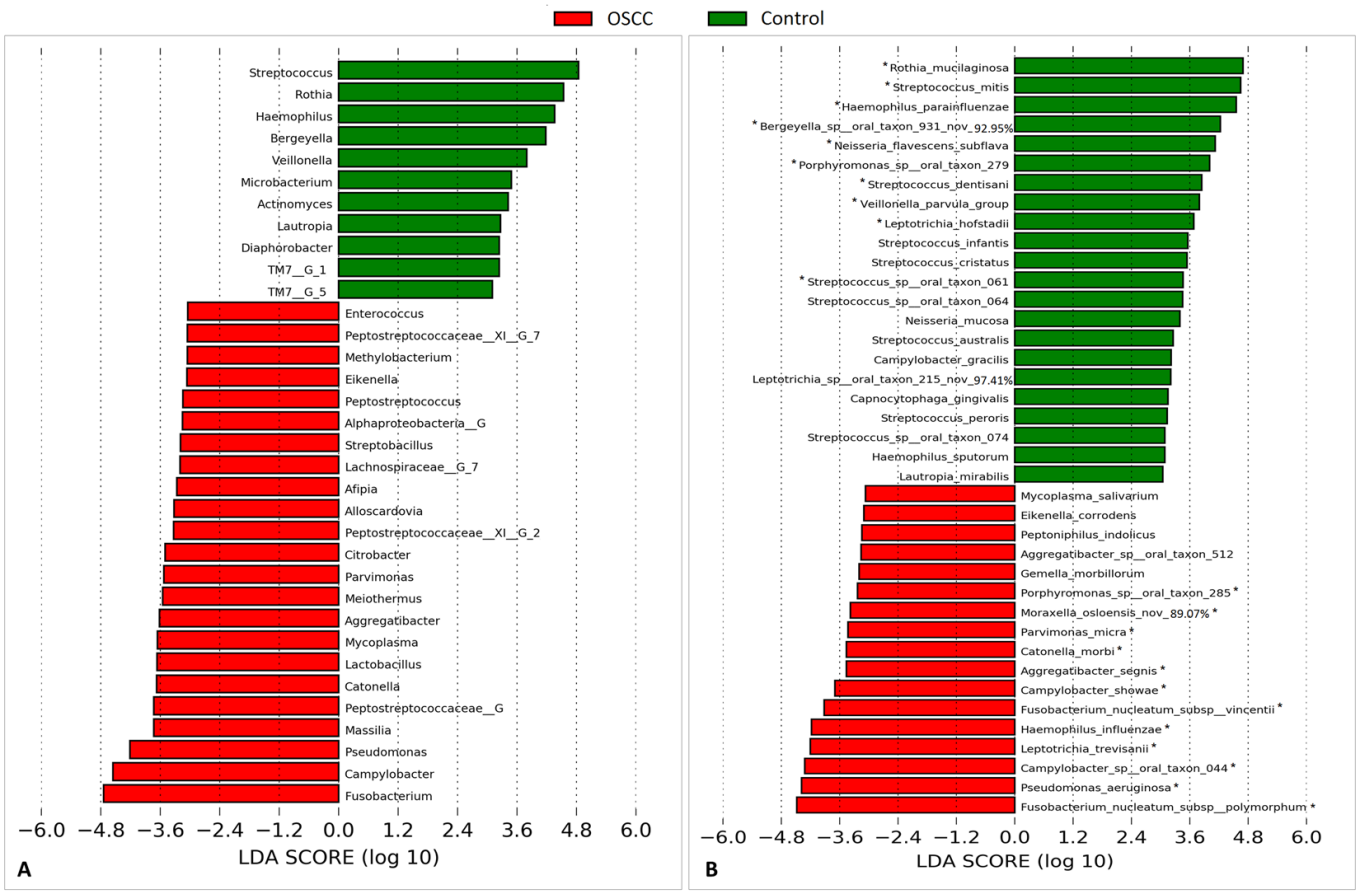
Differentially abundant taxa. Linear Discriminant Analysis Effect Size (LEfSe) analysis showing genera (**A**) and species (**B**) that were significantly differentially abundant between the cases and controls (LDA score ≥ 3). *The difference is also significant by G-test (False discovery rate = 0).

The genera and species with significantly different abundance in the cases and controls are presented in Fig. 4. *Fusobacteria*, *Campylobacter and Pseudomonas* showed the strongest association with OSCC, *while Streptococcus*, *Rothia and Haemophillus* were the most overrepresented genera in the controls. Species-wise, *F*. *nucleatum subsp*. *polymorphum*, *Pseudomonas aeruginosa and Campylobacter sp*. Oral taxon 44 were the most significantly abundant in the tumors, while *S*. *mitis*, *R*. *mucilaginosa* and *H*. *parainfluenzae* were the most associated with the controls. The distribution of these 6 species in each of the study groups, overall and by sample collection site is presented in Supplementary Fig. 1. We elaborate here on *F*. *nucleatum subsp*. *polymorphum* and *P*. *aeruginosa*. The former accounted for more than 10% (and as high as 34%) of the reads in 7 (35%) of the OSCC samples but in only one control sample. Stratifying by sampling site, however, it maintained significant association only with tongue cancer. *P*. *aeruginosa* was identified in 70% of the tumors compared to only 15% in the controls; the abundance in the former reached 23% while it did not exceed 0.05% in the latter. After stratification, the association remained significant for cancer of the tongue and gum.

Species exclusively found in either groups at ≥15% regardless of whether or not they were detected by LEfSe as differentially abundant are listed in Supplementary Table 1. Among these were potentially pathogenic taxa e.g. *Haemophilus influenzae* (30%), *Staphylococcus aureus* (20%), *Bacteroides fragilis* (15%) and *Escherichia coli* (15%).

### Differentially enriched genes and pathways

**Figure 5:**
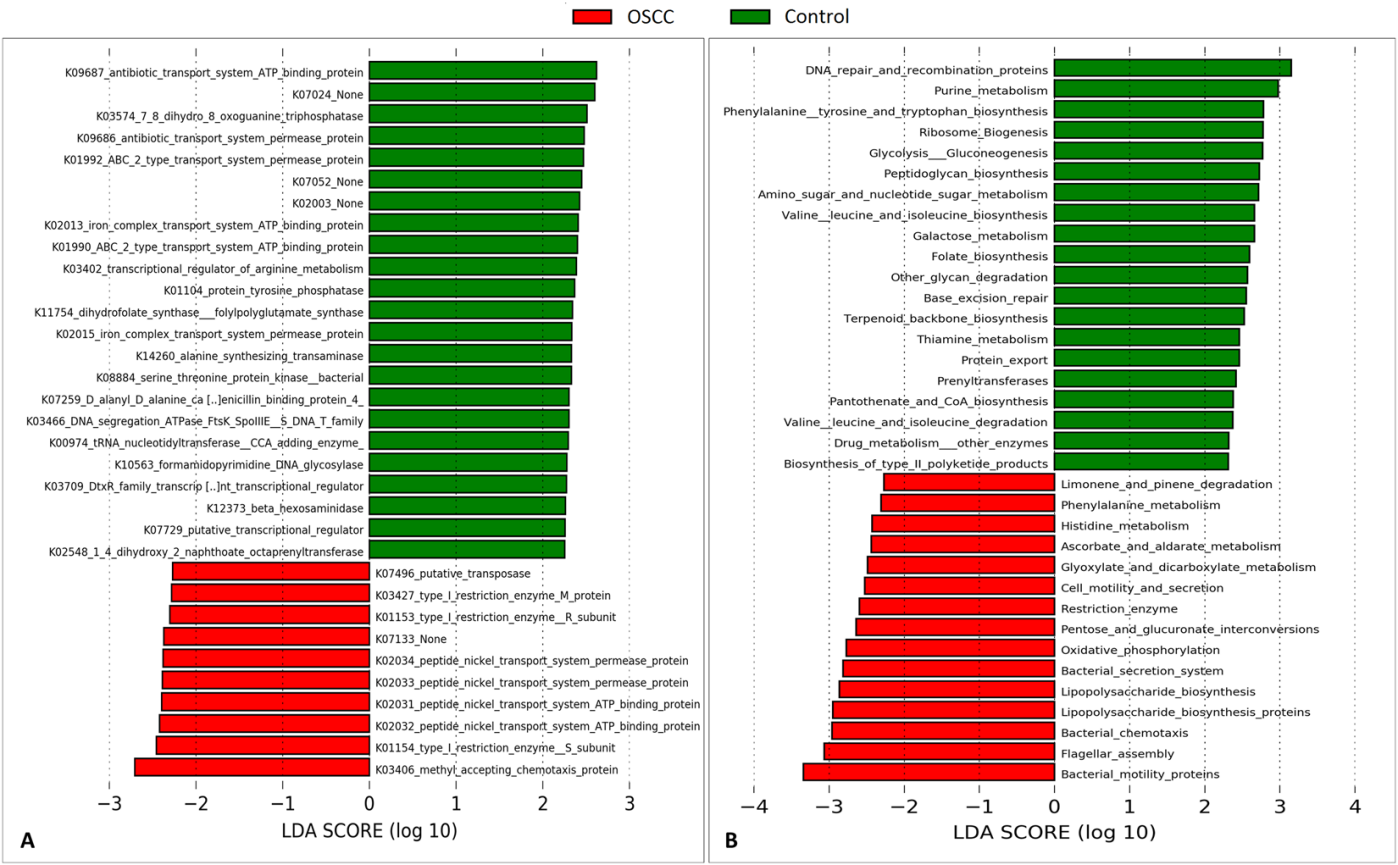
Differentially enriched functions. Linear Discriminant Analysis Effect Size (LEfSe) analysis showing genes (**A**) and pathways (**B**) that were significantly differentially enriched between the cases and controls (LDA score ≥ 2.25).

The microbial genes and pathways enriched in each of the study groups are shown in Fig. 5. At the gene level, genes encoding methyl accepting chemotaxis protein, restriction enzyme subunits and peptide nickel transport system permease and ATP binding proteins were enriched in the cases while those encoding antibiotic transport system permease and ATP binding proteins, 7,8-dihydro-8-oxoguanine-triphosphatase and ABC-2 type transport system permease and ATP binding proteins were the most overrepresented genes in the controls. At the pathway level, genes involved in bacterial mobility, flagellar assembly, bacterial chemotaxis and LPS synthesis were significantly more abundant in the tumor samples, while those involved in DNA repair and combination, purine metabolism, phenylalanine, tyrosine and tryptophan biosynthesis, ribosome biogenesis and glycolysis/gluconeogenesis were the most significantly associated with the controls.

## Discussion

This is the first full-scale study to employ NGS for characterization of bacteria within OSCC tissues to the species level. It is also the first report on the functional potential of the bacteriome associated with OSCC. To maximize reliability of comparisons, the cases and controls were matched for gender, age and sampling site. Theoretically, tissue biopsies from the healthy subjects would have served the best control samples, but that was not possible due to ethical concerns. Instead, epithelium swabs were obtained, which may be viewed as an inevitable study limitation. Unlike previous studies, however, deep swabbing was performed so as to recover within-tissue rather than surface bacteria. Another limitation is that, due to the circumstances of the study, the controls had to be recruited in a setting and geographical location different from that of the cases. However, Jazan is located just across the Northern border of Yemen; in fact, this was a disputed region between Yemen and Saudi Arabia until 2001. In any case, there are no ethnic differences between the two countries. In addition, Jazan is culturally similar to Yemen. Of particular importance and relevance to the study is that OSCC is highly prevalent in both regions^37, 38^ with the major risk factor in common: *shammah* use^24, 39, 40^. Therefore, the difference in frequency of shammah use between the cases and controls is a reflection of the fact that shammah use is associated with OSCC, not because the controls were recruited in a different geographical location. However, the possibility remains that shammah use accounted, at least in part, for the differences in the bacteriome observed between the cases and controls. If so, it may be one mechanism by which shammah use contributes to oral carcinogenesis.

We exploited Illumina’s 2 × 300 bp sequencing chemistry coupled with stringent read stitching and quality-filtering algorithms to generate high quality, full length V1-V3 reads (472–562 bp) and thus maximize the resolution and accuracy of species-level taxonomic assignment obtained with the prioritized BLASTN-based classification pipeline used. The advantages of using this classification algorithm over *de novo* OTU calling and the rationale of prioritizing the reference 16S rRNA sequence databases used have been discussed in previous reports^10, 41^. One study limitation is that only predictive functional analysis was performed using PICRUSt. Although PICRUSt has been demonstrated to produce accurate functional predictions when compared to whole metagenome sequencing^36^, the latter remains the gold standard to confidently characterize the functional attributes of microbial comminutes.

The OSCC and control samples had similar species richness and α-diversity, which is consistent with previous reports in which tissue biopsies or swabs have been analyzed^16, 19^. In contrast, saliva samples obtained from OSCC subjects have been demonstrated in two studies to have significantly lower species richness and α-diversity than those obtained from control subjects^11, 17^, suggesting that salivary bacterial diversity may be used as a marker of OSCC risk. The average number of species per sample detected in this study is a bit higher than that found in our previous pilot study (142 vs. 118), obviously because of the higher sequencing depth here, but remains much less – and thus realistic- compared to the numbers reported in studies employing *de novo* OTU calling which is known to significantly inflate species richness^41^.

Many taxa were found to be differentially abundant between the cases and controls as identified by LEfSe and G-test. *Fusobacterium* was the most significantly abundant genus in the OSCC samples. Consistently, Nagy *et al*.^15^ and Schmidt *et al*.^19^ identified *Fusobacterium* at significantly higher levels in swabs of OSCC lesion surface compared to those of normal mucosa from the same patients. At the species level, however, the current study provides the first epidemiological evidence ever for association of *F*. *nucleatum* with OSCC, substantiating existing evidence on its carcinogenicity. *F*. *nucleatum* has been associated with colorectal carcinoma (CRC)^42, 43^ and demonstrated to promote cellular proliferation and invasion in human epithelium and CRC cell lines^44, 45^ and to enhance progression of OSCC and CRC in animal models^46, 47^. In this study, the association is specifically shown for *F*. *nucleatum subsp*. *polymorphum* and *F*. *nucleatum subsp*. *vincentii*, suggesting there may be differences in the carcinogenicity of this species at the subspecies level, a possibility never explored before.

For the first time, we here report an association between *P*. *aeruginosa* and OSCC. This species has not been linked in the literature to any cancer type. However, there is some recent evidence from *in vitro* studies to suggest a role in carcinogenesis^48^. For example, *P*. *aeruginosa* has been demonstrated to trigger DNA breaks in epithelial cells^49^, which could result in chromosomal instability. *P*. *aeruginosa* possesses structures, e.g. lipopolysaccharides (LPS) and flagella, and cytotoxins (e.g. ExoU) with potent proinflammatory activity that results in recruitment of neutrophils via activation of NF-κB signaling pathway^50, 51^. This is relevant because inflammation is accepted to play an important role in carcinogenesis. Furthermore, *P*. *aeruginosa* secrets factor LasI that disrupts adherens junctions and reduces expression of E-cadherin, a molecule known to serve antagonistic function against cellular invasion and metastasis^48^. Whether *P*. *aeruginosa* plays a role in initiation or/and progression of OSCC thus warrants further investigation.

*Streptococcus* and *Rothia* were the most significantly associated genera with the controls, which is consistent with findings from the study by Schmidt *et al*.^19^ in which surface swabs were analyzed. In contradiction, Pushalkar *et al*.^17^ and Guerrero-Preston *et al*.^11^ found these genera to be more abundant in the saliva samples of OSCC. This, along with the differences in species richness and diversity for tissue biopsies and saliva described above, suggests that bacterial associations with OSCC dramatically differ by, and should thus be differently interpreted based on, the type of sample analyzed. In line with this, *S*. *mitis* was found here as well as in the study by Pushalkar *et al*.^16^ to be overrepresented in the control samples, while it was shown by Mager *et al*.^13^ to be more abundant in saliva samples from OSCC patients. *R*. *mucilaginosa* and *H*. *parainfluenzae* were among the top taxa showing association with health in this study. Consistently, Pushalkar *et al*. detected *R*. *mucilaginosa* much more frequently in their non-tumor samples. In addition, both species have been recently reported as members of the healthy core oral bacteriome^41^.

The bacteriome predicted functions found to be enriched in the OSCC samples in this study are strikingly similar to those identified very recently in association with chronic periodontitis^52^, emphasizing they are proinflammatory in nature. Indeed, bacterial flagella and LPS are potent inflammatory structures. The latter in particular has been found to induce cancer-promoting inflammatory reactions. For example, LPS has been demonstrated to promote invasiveness of pancreatic cancer by activation of the TLR/MyD88/NF- NF-κB pathway^53^, to facilitate lung metastasis in a breast cancer via the prostaglandin E2-EP2 pathway^54^ and to increase liver metastasis of human CRC by stimulation of toll receptor TRL4^55^. Flagella associated with *P*. *aeruginosa* are known to induce inflammation by activation of the NF-κB^48^; although, there is no evidence linking this to carcinogenesis directly, the possibility cannot be excluded. Bacterial chemotaxis also seems to play an important role in cancer-related inflammation. Studies on H. pylori, for example, show that mutants defective in chemotaxis induce less inflammation than the wild type^56^. Overall, therefore, the bacteriome associated with OSCC can functionally be described as “inflammatory” which is a very important finding given the established role of inflammation in cancer. However, whole metagenome sequencing in a more extensive and independent functional study is required to confirm and explore these findings further.

In conclusion, a distinct bacteriome, compositionally and functionally, is associated with OSCC in these Yemeni patients. This study provides the first epidemiological evidence for association of *F*. *nucleatum* and *P*. *aeruginosa* with OSCC. It also suggests there may be some variation in carcinogenicity of *F*. *nucleatum* subspecies. At the functional level, the bacteriome enriched in OSCC can be described as “inflammatory”. Exploring the role of differentially abundant taxa and pathways identified in the development and/or progression of OSCC is warranted.

## Electronic supplementary material

Supplementary materials

Dataset 1

Dataset 2

Dataset 3
