# Supplementary material for: Inflammatory bacteriome featuring Fusobacterium nucleatum and Pseudomonas aeruginosa identified in association with oral squamous cell carcinoma
Source: Sci Rep. 2017 May 12;7:1834. doi: 10.1038/s41598-017-02079-3 (PMC5431832; doi:10.1038/s41598-017-02079-3)
Supplement: Supplementary file 1 — Supplementary materials [file 41598_2017_2079_MOESM1_ESM.pdf]

Inflammatory bacteriome featuring *Fusobacterium nucleatum* and *Pseudomonas aeruginosa* identified in association with oral squamous cell carcinoma

Nezar Noor Al-hebshi, Akram Thabet Nasher, Mohamed Yousef Maryoud , Husham E. Homeida, Tsute Chen, Ali Mohamed Idris and Newell W Johnson

Supplementary materials

Legends

Supplementary Dataset 1. Abundances and detection frequencies of phyla (ranked from highest to lowest) in each of the samples and across the study groups/population.

Supplementary Dataset 2. Abundances and detection frequencies of genera (ranked from highest to lowest) in each of the samples and across the study groups/population.

Supplementary Dataset 3. Abundances and detection frequencies of species (ranked from highest to lowest) in each of the samples and across the study groups/population.

Supplementary Figure 1. Boxplots showing the distribution of top differentially abundant species in each of the study groups, overall (left) and by collection site (right). The error bars represent data within 1.5 interquartile range above the third quartile and below the first quartile. Hollow circles and stars are outliers. Colored circle denote species for which the differences were significant, Mann-Whitney test

Supplementary Table 1. List of taxa exclusively identified in either group at prevalence $\geq$ 15%.

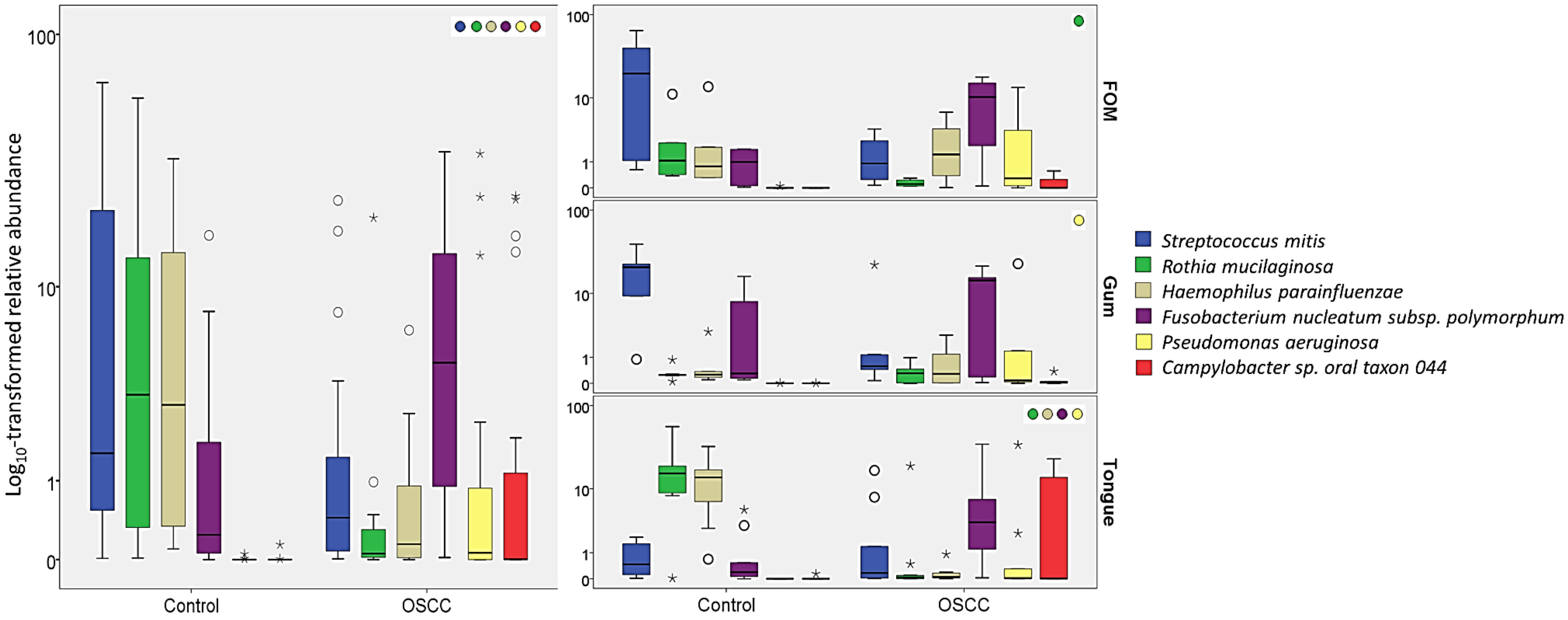

Supplementary Figure 1. Boxplots showing the distribution of top differentially abundant species in each of the study groups, overall (left) and by collection site (right). The error bars represent data within 1.5 interquartile range above the third quartile and below the first quartile. Hollow circles and stars are outliers. Colored circle denote species for which the differences were significant, Mann-Whitney test.

Table S1. List of taxa exclusively identified in either group at prevalence ≥ 15%

Exclusively in OSCC	%	Exclusively in Controls	%
Alloscardovia omnicolens	35%	*Bergeyella sp.* oral taxon *931*	50%
Atopobium sp. oral taxon *199*	30%	*Bergeyella sp.* oral taxon *931 nov 92.95%*	40%
Lachnospiraceae [G-7] sp. oral taxon *163*	30%	*Haemophilus parainfluenzae nov 97.14%*	40%
Oribacterium sp. oral taxon *102*	30%	*Veillonella sp.* oral taxon *C09*	35%
Streptobacillus hongkongensis	30%	*Porphyromonas pasteri*	30%
Haemophilus influenzae	30%	*Ruminococcaceae [G1] sp.* oral taxon *075 nov 91.53%*	30%
Peptococcus sp. oral taxon *167*	25%	*Neisseria oralis*	30%
Peptostreptococcaceae [XI][G-4] 103/369	25%	*Granulicatella adiacens nov 95.99%*	25%
Moraxella osloensis nov 89.07%	25%	*Streptococcus peroris*	25%
Porphyromonas uenonis	20%	*Rothia mucilaginosa nov 89.14%*	20%
Meiothermus timidus	20%	*Selenomonas artemidis*	20%
Staphylococcus aureus	20%	*Diaphorobacter nitroreducens* oral taxon *A07*	20%
Peptoniphilus indolicus	20%	*Actinomyces sp.* oral taxon *175 nov 97.96%*	15%
Peptostreptococcaceae [G] sp. oral taxon *B61*	20%	*Actinomyces sp.* oral taxon *178*	15%
Methylobacterium rhodesianum	20%	*Actinomycetales [G] sp.* oral taxon *C05*	15%
Neisseria sp. oral taxon *018*	20%	*Bifidobacterium dentium*	15%
Dietzia cinnamea nov 93.21%	15%	*Prevotella aurantiaca*	15%
Bifidobacterium longum	15%	*Prevotella melaninogenica nov 96.55%*	15%
Janibacter indicus	15%	*Streptococcus sp.* oral taxon *B66*	15%
Bacteroides fragilis	15%	*GN02 [G1] sp.* oral taxon *872*	15%
Capnocytophaga sp. oral taxon *901*	15%	*Betaproteobacteria [G] sp.* oral taxon *B96*	15%
Enterococcus avium	15%	*Campylobacter sp.* oral taxon *G43*	15%
Lactobacillus salivarius	15%	*TM7 [G1] sp.* oral taxon *349 nov 97.79%*	15%
Butyrivibrio sp. oral taxon *080*	15%		
Eubacterium yurii nov 90.91%	15%		
Bulleidia extructa	15%		
Selenomonas sp. oral taxon *134*	15%		
Fusobacterium gonidiaformans	15%		
Fusobacterium multispecies spp9 2	15%		
Leptotrichia sp. oral taxon *221 nov 90.45%*	15%		
Alphaproteobacteria [G] sp. oral taxon *A28 nov 84.07%*	15%		
Alphaproteobacteria [G] sp. oral taxon *C96*	15%		
Afipia broomeae	15%		
Achromobacter xylosoxidans	15%		
Alcaligenes sp. str. CO14	15%		
Delftia sp. oral taxon *A59*	15%		
Ottowia sp. oral taxon *894 nov 97.31%*	15%		
Cupriavidus metallidurans	15%		
Massilia timonae	15%		
Neisseria sp. oral taxon *018 nov 97.96%*	15%		

Citrobacter koseri	15%		
Escherichia coli	15%		
Aggregatibacter paraphrophilus	15%		
Acinetobacter baumannii nov 96.09%	15%		
Moraxella lacunata	15%		
